# Oral health effects of ecstasy (MDMA) and methamphetamine: a narrative review

**DOI:** 10.3389/froh.2025.1645445

**Published:** 2025-09-12

**Authors:** Lily-Rose Newell, Kevin-John Fouillen, Marie Orliaguet, Johanna Kichenin, Sylvie Boisramé

**Affiliations:** 1 Brest Faculty of Dentistry, Université de Bretagne Occidentale, Brest, France; 2 Department of Oral Surgery, Oral Medicine and Dentistry, CHU de Brest, Brest, France; 3 ER 7479, SPURBO, Université de Bretagne Occidentale, Brest, France; 4 UR 4685, Laboratoire Interactions Epithéliums Neurones (LIEN), Université de Bretagne Occidentale, Brest, France; 5 UMR 1078, Laboratoire GGB, Brest, France

**Keywords:** MDMA, ecstasy, 3,4-methylenedioxymethamphetamine, Methamphetamine, drug abuse and oral cavity, xerostomia, meth mouth

## Abstract

This review aims to explore the impact of 3,4 methylenedioxymethamphetamine (MDMA), commonly known as ecstasy, on oral health, while also drawing comparisons with methamphetamine (MA) due to their pharmacological similarities and overlapping oral manifestations. MDMA is a psychostimulant derivative of amphetamine (AM) with empathogenic and hallucinogenic properties, widely consumed, especially among young adults. Its pharmacological effects lead to both acute and long-term systemic consequences. Among its oral manifestations, xerostomia is notably prevalent and strongly associated with increased intake of sugary beverages, contributing to heightened risks of carious lesions, tooth wear and periodontal disease. Bruxism and jaw clenching, frequent during MDMA use, are implicated in temporomandibular joint dysfunction and can lead to significant tooth wear lesions. This review also discusses periodontitis prevalence, often linked to poor oral hygiene, poly-drug use, and behavioral changes. Additionally, cases of soft tissue damage and unique patterns of decay have been documented. These findings highlight the need for dental professionals to recognize oral health issues associated with MDMA, MA and polydrug use.

## 1 Introduction

Drugs can be classified into several categories, including stimulants, depressants, hallucinogens and narcotics (1). Stimulants, such as caffeine and amphetamines, increase alertness and activity levels. Depressants, such as alcohol and benzodiazepines, slow down brain function and can induce relaxation or sedation. Hallucinogens, including LSD and psilocybin, alter perception, mood, and consciousness. Narcotics, particularly opioids like heroin and prescription painkillers, relieve pain but also have a high potential for addiction. Other classifications may consider factors such as legality, addictive potential, and medical use. Psychostimulants [cocaine, amphetamines (AMPH), and cathinones] are sympathomimetic substances that exert effects on both the central nervous system (CNS) and the peripheral nervous system (PNS), akin to those elicited by adrenaline and noradrenaline (2).

MDMA (3,4 methylenedioxymethamphetamine), an amphetamine derivative with stimulant and hallucinogenic properties, was first synthesized in 1912 by the pharmaceutical company Merck. However, it did not achieve widespread popularity until the late 1970s, particularly within the dance and rave subcultures (3). In France, approximately 8.2% of individuals aged 18–64 had experimented with MDMA in 2022. The age groups most prominently associated with experimentation were 25 to 34-year-olds (13.8% experimenters) and 35–44 years olds (11.6%) (4).

As of 2021, in France, approximately 6% of deaths attributable to substance abuse were linked to amphetamines or MDMA, accounting for 29 fatalities. This proportion has remained relatively stable since 2012, as per data from the DRAMES Survey 2019, CEIP-A Grenoble-ANSM (5).

MDMA is available in various forms, including tablets (commonly known as ecstasy), crystals, and powder, typically consumed orally. It is occasionally dissolved in beverages or ingested via a “parachute” (where the substance is wrapped in rolling paper), but may also be inhaled nasaly or less commonly smoked or injected (4). In comparison, methamphetamine (MA), is used via routes other than oral, usually smoked and intravenous, which increases the likelihood of abuse and other deleterious effects (6).

MA use is typically chronic and compulsive, involving daily or high-frequency intake and strongly associated with dependence and high-risk behaviors (7–10).

Consumption is typically driven by the desire to experience sensations of euphoria and well-being along with empathogenic and entactogenic effects giving an enhanced resistance to fatigue. At higher doses, MDMA can induce hallucinogenic effects, potentially leading to alterations in sensory perception (7).

Although MDMA and methamphetamine (MA) share several effects, they are distinct substances in terms of chemical structure, mechanisms of action and patterns of use (6). MA stimulates release and blocks reuptake of dopamine, norepinephrine and serotonin, leading to neurodegeneration and neurotoxicity (11) while MDMA exerts a stronger effect on serotonin release (6) known as serotonin syndrom (12–14). It also inhibits the synaptic reuptake transporters resulting in increased levels of the neurotransmitters within the synaptic cleft, which in turn affects mood, energy levels, appetite, trust, sexual activity, emotions and sleep (15).

When an ecstasy tablet is taken orally, its effects begin within 20–60 min, peak at 2 h, and last for 4–6 h. The plasma half-life of MDMA ranges from 6 to 9 h. Approximately 80% of it is metabolized in the liver by the enzyme CYP2D6, while the remaining 20% is excreted unchanged in urine, detectable for 2–3 days after ingestion. Ecstasy is also excreted in other bodily fluids like tears, saliva, sweat, and breast milk (14).

The immediate behavioral impacts, similar between MA and MDMA, encompass sympathomimetic activation: bronchodilation, mydriasis, feelings of euphoria, enhanced alertness and motivation, difficulty sleeping, reduced appetite, elevated breathing and body temperature, enhanced sensory experiences, and a stronger sense of connection with others (3). Over time, these can lead to psychological addiction and dependency rather than physical addiction. Long-term effects may include insomnia, hypertension, tachycardia, hyperthermia, restlessness, continued hyperactivity, decreased appetite and subsequent weight loss, tremors, and repetitive movements. Paranoia is a lasting consequence that may persist for years after cessation, potentially exacerbated by auditory and visual hallucinations (8, 14, 16).

Additionally, the drug's systemic effects can result in various oral manifestations, including bruxism, jaw clenching, xerostomia and tooth decay, commonly referred to as “meth mouth” in the case of MA consumption, as well as ulcerations.

The primary objective of this review is to assess the effects of MDMA on the oral cavity, and to compare these results with those observed in MA users and polydrug users.

## 2 Materials and methods

### 2.1 Research strategy

A comprehensive analysis of the literature was performed using databases such as PubMed and Web of Science.

The computerized literature search was performed using the following terms: “MDMA and oral cavity” “MDMA and teeth” “MDMA and periodontitis” “Ecstasy and oral cavity” “Ecstasy and teeth” “Ecstasy and periodontitis” “MDMA and mouth” “Ecstasy and mouth”.

### 2.2 Selection criteria

No restrictions on the origin of the study or the year of publication interfered in the article selection.

Studies were included based on the following inclusion criteria: studies reporting the impact of MDMA use on the oral cavity. MA related studies were also included for comparative purposes, given the frequent overlap in polydrug use. The selection was refined according to the following exclusion criteria: - Studies focusing on the impact of substances other than MDMA, MA. - Studies not addressing oral health outcomes or conditions of the oral cavity. - Articles published in languages other than English or French.

Duplicates were removed, then titles were screened for relevance. Abstracts were screened by one of the authors (LRN); a second author (SB) double-checked the article selection process. In cases of disagreement, LRN and SB consulted each other to come to a consensus.

### 2.3 Data extraction

For each included study, the full text was reviewed to extract all the information, namely, side effects impacting the oral cavity: teeth, periodont, soft tissues, saliva.

## 3 Results

### 3.1 Study selection

**Figure 1:**
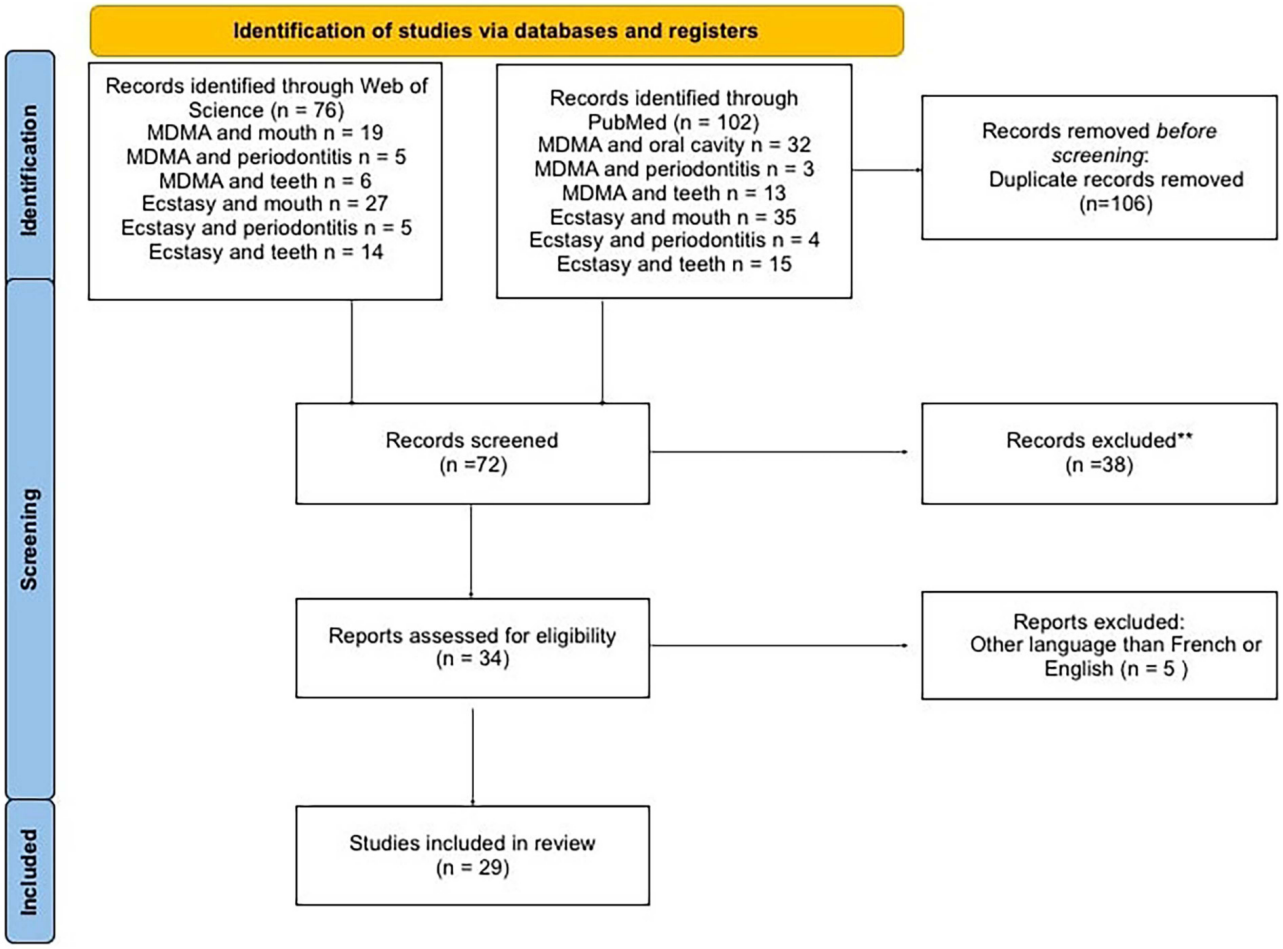
Flowchart of selected articles.

The initial search found a total of 178 articles. After duplicates were removed and the records were screened, 29 articles were included for data extraction (Figure 1).

### 3.2 Study characteristics

To facilitate the comprehension of the 29 included articles, we separated the results into 5 subgroups according to the subjects they dealt with (saliva, teeth, temporomandibular joint, periodontium and soft tissues) (Table 1).

**Table 1:** Overview of the included studies (in alphabetic order by author).

Author/Year	Country	Study design	Population/Sample size	Substance(s) investigated	Oral health outcomes	Key findings
Ahmed et al. (2007) (17)	UK	Case Report	1 patient	MDMA	Mucosal oedema	MDMA use induced widespread oral and oropharyngeal mucosal oedema.
Allott et al. (2009) (13)	Australia	Controlled Study	9 Male & 11 female MDMA polydrug users	MDMA	Neuroendocrine & oral health	Reported serotonergic changes linked to oral manifestations.
Arrue et al. (2004) (18)	Spain	Experimental (rats)	Animal model	MDMA	Jaw reflex	MDMA altered jaw-opening reflex and *α*2-adrenoceptors regulation.
Baylen & Rosenberg (2006) (16)	USA	Review	NA	MDMA	Acute oral effects	Summarized acute subjective and somatic effects, including bruxism.
Biancardi et al. (2019) (19)	Brazil	Case reports	2 MDMA users	MDMA	Oral mucosa lesions	Oral ulcerations found mucosal irritation and inflammation associated with MDMA.
Brand et al. (2008) (8)	Netherlands	Review	NA	MDMA	Xerostomia, bruxism	MDMA linked to xerostomia, dental erosion, jaw clenching, and tooth wear.
Brazier et al. (2003) (20)	UK	Case Report	1 patient	MDMA	Periodontitis, ulcers	Ecstasy-related periodontitis and mucosal ulceration observed.
Dinis-Oliveira et al. (2010) (21)	Portugal	Case Report	1 patient	MDMA	Bruxism	Severe bruxism after MDMA abuse.
Duxbury (1993) (22)	UK	Review	NA	MDMA	General oral effects	Highlighted dental implications of ecstasy use.
Fratto & Manzon (2014) (23)	Italy	Review	NA	Psychotropic drugs	Dental disease	Oral effects of stimulants.
Murray & Wilson (1998) (24)	UK	Case Report	1 patient	MDMA	Tooth wear	Ecstasy use associated with accelerated tooth wear.
Maloney (2014) (12)	USA	Review	NA	MDMA	Dental implications	Discussed the importance of recognizing ecstasy's impact in dental practice.
McGrath & Chan (2005) (25)	UK	Cross-sectional survey	119 poly drug abusers	Various drugs including MDMA	Oral health sensations	Reported xerostomia, bruxism, and mucosal ulcerations.
Milosevic et al. (1999) (26)	UK	Cross-sectional study	30 ecstasy users and 28 non ecstasy users	MDMA	Tooth wear	Ecstasy use associated with significant tooth surface loss.
Mudhar & Agarwala (2021) (27)	UK	Case Report	1 patient	MDMA	Oral mutilation	Severe oral injury following MDMA use.
Nixon et al. (2002) (28)	UK	Comparative clinical study	13 amphetamine like drug users and 13 non drug users	MDMA+ others	Tooth surface loss	Drug use contributed to enamel erosion and wear.
Nugent et al. (2017) (29)	UK	Case Report	1 patient	MDMA	Severe mutilation	Reported oral mutilation linked to ecstasy.
Paz-Ramos et al. (2023) (3)	Mexico	Review	NA	Amphetamine-type stimulants	Oral health risks	Provided updated insights on ATS effects, including oral consequences.
Peroutka (1988) (30)	USA	Survey	100 Recreational MDMA users	MDMA	Subjective effects	Included bruxism, xerostomia among reported symptoms.
Quaranta et al. (2022) (14)	Italy	Review	NA	Illegal drugs incl. MDMA	Periodontal health	Ecstasy use increases the risk of periodontitis.
Redfearn et al. (1998) (31)	UK	Cross-sectional study	30 ecstasy users and 28 non ecstasy users	MDMA	Tooth wear	Significant association between MDMA use and excessive tooth wear.
Richards & Brofeldt (2000) (32)	USA	Prospected case study	43 MA users	MA	Tooth wear	MA use strongly linked to severe tooth wear.
Rhodus (2005) (33)	USA	Review	NA	MA	“Meth mouth” and ecstasy	Documented rampant caries and tooth oral decay in meth users.
Shaner et al. (2006) (9)	USA	Case Report	MA abuser	MA	Rampant caries	Severe decay (“meth mouth”) observed and xerostomia and bruxism observed in ecstasy users.
Shekarchizadeh et al. (2013) (34)	Iran	Review	NA	Multiple drugs	Oral manifestations	Summarized oral health effects of drug abuse in ecstasy users: Dental erosion, high amounts of acidic sugary drinks, xerostomia, ulcers, vestibular swelling, edema, and necrosis.
Shetty et al. (2015) (10)	USA	Cross-sectional study	571 MA users	MA	Caries, periodontitis	High prevalence of caries and periodontitis in meth users.
Valadas et al. (2020) (35)	Brazil	Review	NA	Multiple drugs	Oral manifestations	Review highlighted oral lesions and decay in drug abusers.
Van Kempen et al. (2022) (36)	Netherlands	Cross-sectional study	Ecstasy users vs. controls	MDMA	Periodontitis, caries, xerostomia	Higher prevalence of periodontitis, caries, and dry mouth in ecstasy users.
Yeh et al. (2022) (15)	Taiwan	Experimental (primate)	Animal model	MDMA	Long-term serotonergic effects	Potential implications for oral health via serotonergic pathways.

ATS, amphetamine-type stimulants; MA, methamphetamine; MDMA, 3,4-Methylenedioxymethamphetamine; NA, not applicable.

### 3.3 Impact on saliva

Seven studies have documented the subjective adverse effect of dry mouth when consuming MDMA (21, 25, 27, 31, 35–37).

For instance, a 2004 study (25) involving 119 poly-drug users, of whom 80% used MA and 58% used MDMA, reported that nearly all participants (95%) experienced “dryness” of the mouth. Another study (9) involving young drug addicts found a 73% reduction in stimulated parotid salivary flow among MA users and a 59% reduction among subjects abusing both MA and cannabis.

A study by Van Kempen et al. (36), which surveyed 149 recreational ecstasy users, found that 44.3% of participants also reported using other psychoactive substances, with cannabis being the most common. Notably, the co-use of cannabis appeared to intensify xerostomia: while only 16.7% of ecstasy-only users reported experiencing dry mouth, this prevalence increased to 42.3% among those who also used cannabis. This xerostomia can persist for up to 48 h after consuming ecstasy (8, 27, 36), particularly pronounced in females.

Interestingly, the frequency of ecstasy use does not appear to significantly influence the occurrence of dry mouth. In a study comparing occasional users (one to three times) with frequent users, no statistical difference in the prevalence of self-reported xerostomia was found (36). Instead, the incidence of dry mouth appears to be dose-dependent. For example, among healthy volunteers, dry mouth or throat was reported by 25% two hours after administering 0.5 mg MDMA/kg, increasing to 88% after a dose of 1.5 mg/kg. Furthermore, xerostomia persisted longer after the higher dose (8, 16).

Other factors contributing to xerostomia may include heightened physical activity during ecstasy use (16) and concurrent use of other recreational psychoactive drugs. Ecstasy users often consume higher amounts of prescribed psychotropic medication, which could further exacerbate xerostomia (23).

While dry mouth is commonly described as a subjective adverse effect, hyposalivation may also occur. MDMA has an affinity for peripheral noradrenergic neurotransmission via α-2-adrenergic receptors, potentially inducing salivary hypofunction (36).

To alleviate dry mouth, users often consume large volumes of fluids during a trip, with carbonated drinks being particularly popular (31). Ecstasy users have a weekly consumption of 10 units of fizzy drinks compared to 6 units in non users (31).

### 3.4 Impact on teeth

#### 3.4.1 Dental caries

Although MA and MDMA are distinct substances, their oral health consequences often overlap, particularly in terms of dental caries.

In the study by V.Shetty (10), older MA users (>30 years old) exhibited higher DMFT (decayed, missing and filled teeth) scores, with 97% reporting a history of dental caries and 59% having untreated dental caries. Within the MA users cohort, 31% had six or more missing teeth, compared to 8.5% in the general population.

**Figure 2:**
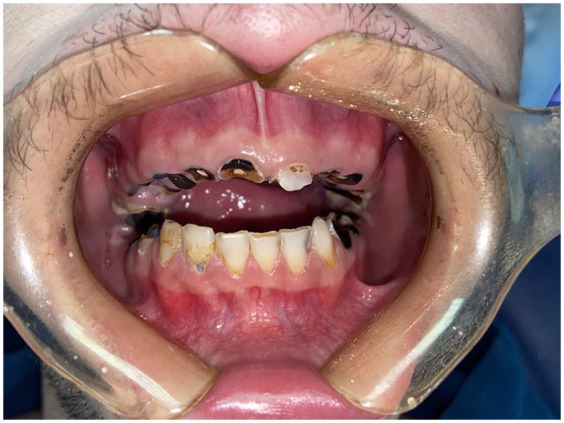
Clinical presentation of a 22 year old male patient using MDMA for 3 years and still consuming at the time of presentation combined with a 20 cigarettes per day habit since he was 14 years old.

**Figure 3:**
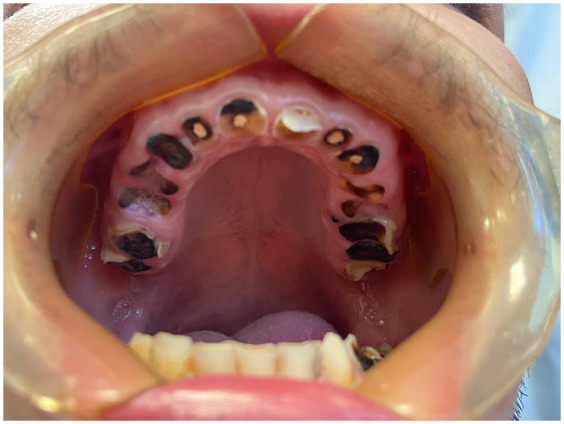
Clinical presentation of the same patient highlighting destruction of all the coronal portion of the maxillary teeth.

Similar to xerostomia, a dose-response relationship between higher levels of MA use and increased rates of tooth decay exists. This decay pattern, illustrated by the case report of J.W Shaner (9) is characteristic: it initially affects the smooth surface on the vestibular side of the posterior teeth and the areas between adjacent anterior teeth, and eventually leads to the total destruction of the tooth's coronal portion (Figures 2, 3).

A cross-sectional study (10) on MA users analyzed the decay patterns, revealing that mandibular first molars were the most frequently missing teeth/surface among approximately 40% of the sample (552 subjects), with a left/right symmetry. However, a higher prevalence of surface decay on the distal surface of the maxillary right premolar, not present at the same prevalence on the corresponding left premolar was noted.

#### 3.4.2 Tooth wear

**Figure 4:**
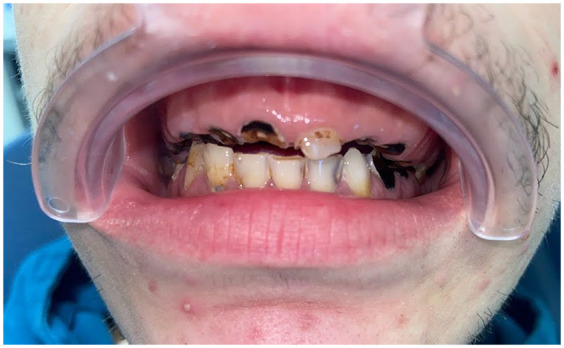
Clinical presentation of the same patient showing the total destruction of the posterior teeth with only preservation of the anterior mandibular teeth.

The relationship between MDMA consumption and tooth wear lesions such as erosion and attrition was first proposed in the early 1990s (22, 24). They introduced the hypothesis that ecstasy use could lead to significant dental wear. Their conclusions were supported by a 1988 study (30) involving 29 volunteers who received recreational doses of MDMA (75–150 mg). The study found that 22 participants experienced bruxism or trismus, 9 reported nausea, and 28 experienced appetite suppression effects strongly associated with increased muscle tension and jaw clenching. P.J Redfearn tested this hypothesis in 1998 (31) by comparing tooth wear in a group of ecstasy users (30 subjects) with a comparison group of 28 similarly aged controls. The study revealed that 89% of the consumers admitted to clenching their teeth, leading to greater wear on the posterior teeth than the anterior teeth (Figure 4).

Tooth wear lesions extending through the enamel into the underlying bone was observed in 60% of ecstasy users, compared to only 11% of non users (8). The overall mean tooth wear lesions score, at 0.63, was significantly higher in consumers compared to the mean score of 0.16 in non users (*p* < 0.001). Tooth wear lesions were predominantly found in the molar regions of both upper and lower arches (55% average) and minor incisal wear was noted (26, 28).

Interestingly, different patterns of tooth wear lesions have been reported in MA users (32). Johan R. Richards studied tooth wear lesion severity among MA users based on their route of drug administration. His study led to the result that patients who regularly snorted MA had higher scores for the anterior maxillary teeth than patients who injected, smoked, or ingested MA (*p* = 0.005) supported by a clinical case of a 42 years old male who reported snorting MA on a weekly basis for several years (32).

### 3.5 Impact on temporomandibular joint

Aurora Arrue and her team (18) investigated the effects of intravenously administered MDMA on the digastric electromyographic on responses elicited by orofacial electrical stimulation in the rat by studying the jaw opening reflex (JOR) and the sensitivity of the α-2-adrenoceptor that inhibits de JOR to regulate it. Administration of a single or repeated dose of MDMA in rats induced a partial inhibition of the JOR.

In a study of 2005 (25), 75% of the ecstasy consumers felt like chewing something, 50% noticed they had the habit of grinding or clenching their teeth together and 56% felt pain and tenderness in jaw muscle or jaw joint and over a quarter felt their “joints clicked or popped when eating or opening their mouth joint”. Consumers try to reduce the effects of these movements by using chewing gum, lollipops and/or pacifiers (36).

### 3.6 Impact on the periodontium

In a study comparing a group of ecstasy users with non ecstasy users of 2022 (36) the authors found a significant difference in the presence of periodontitis between the two groups: Periodontitis is more than two times more frequent in the ecstasy user group than in the control group. Ecstasy users also reported less frequent tooth brushing, reduced interdental cleaning, and higher consumption of cigarettes and other substances (36).

A case report (9) of a 25 year old male with a 7 year history of daily use of MA, cannabis and alcohol who overlooked oral hygiene when high on drugs which led to a plaque index of 95% due to neglecting oral hygiene while under the influence of drugs.

Another study (10) supported these findings, 37% of adults aged 35–49 in the US general population have total periodontitis whereas over 89% of the MA users group presented this condition (10).

In one case (20) a periodontal lesion has been found after a 15 year old patient reported that he had used ecstasy as a recreational drug by storing the drug in the upper anterior labial vestibule adjacent to the site of periodontal destruction.

### 3.7 Impact on soft tissues

Bruxism, tooth wear lesions, xerostomia and oral ulcerations remain the most common oral manifestations (8, 16, 19, 25). Furthermore, mucosal involvements such as ulcers, vestibular swelling, edema and necrosis, either alone or after injury (like biting cheeks) have been case-reported in ecstasy users (17, 19, 20, 25).

A 21 year old female (19) presented multiple oral ulcerations and severe oral pain lasting 3 days after the use of powdered ecstasy dissolved in water. A final diagnosis of oral mucositis was established and a complete remission of the oral lesions was observed 30 days after the symptomatic treatment with analgesic and corticosteroid. Furthermore this case showed a clinical pattern suggestive of morsicatio associated with areas of ulcers on the bilateral buccal mucosa, probably linked to the bruxism.

This bruxism can lead to an extreme situation seen in an 18 year old female (29) who presented an extensive tissue loss of her lower lip after ingestion of MDMA and self-inflicted trauma due to involuntary mastication.

A patient (17) experienced sudden and extensive swelling around and inside the mouth shortly after ingesting a single ecstasy tablet. The swelling affected the upper and lower lip tissue, both sides of the cheek tissue, the dorsum of the tongue, and the areas around the tonsils. The swelling appeared greyish-white and did not show signs of ulcers or discharge. Treatment involved corticosteroids, antibiotics, and chlorhexidine mouthwash. Within ten days, the swelling had completely resolved. A similar case was found in a 20 year old woman (37).

## 4 Discussion

Saliva plays a crucial role in maintaining oral health (8, 29). It neutralizes acids, facilitates enamel remineralization and removes food particles, thereby reducing the risk of tooth decay. Furthermore, it contributes to oral health by regulating the pH balance in the mouth, which helps prevent acidic conditions that can lead to tooth enamel erosion and tooth decay. It also forms a protective coating on teeth, mitigating the risk of cavities. The high prevalence of xerostomia in MDMA and MA users, compounded by hyposalivation in some cases, compromises these protective functions.

Xerostomia is frequently dose- dependent and can be intensified by co-use with cannabis or psychotropic medication. Furthermore, the preference for carbonated beverages to counteract xerostomia introduces additional erosive and cariogenic risks, linking MDMA-related salivary dysfunction to increased likelihood of caries and enamel erosion. These findings underscore the multifactorial nature of xerostomia in this population, where both drug-induced physiological changes and behavioral factors contribute to oral health deterioration.

The primary mechanism for decay and periodontal disease seems to stem from a combination of factors including hyposalivation, frequent consumption of carbonated soft drinks, extremely high dental plaque levels (95% in the case report), and inadequate oral hygiene practices (9).

The carious lesions rate among MA abusers is reported to be four times higher than among non-users, leading to the term “meth mouth” coined by the press. Although this term is not as pronounced among MDMA users, they may still present elevated caries risk and early enamel damage due to shared behavioral and physiological risk factors.

Indeed, MDMA users generally present with a less severe manifestation, studies suggest that the underlying mechanisms may be similar (8, 19, 23, 29). For instance, 25% of MDMA users report brushing their teeth fewer than two times per day, compared to 15% in non-users.

Although, the extensive tooth decay seen in MA users has occasionally been erroneously attributed to the drug's “acidic” properties (9). The main organisms involved in this infectious process are typically categorized as *Streptococcus mutans*. The cavity development process involves acid-producing bacteria, inadequate oral hygiene allowing bacterial plaque buildup beyond a cariogenic threshold, frequent exposure to refined carbohydrates metabolized by *S. mutans* in plaque to produce acids, and insufficient saliva, which normally helps buffer and pH drop at the enamel-plaque interface (9).

Unlike the stereotypical “meth mouth” pattern characterized by severe and rapidly progressing carious lesions, affecting the smooth surfaces and interproximal arias of anterior teeth (9, 33), carious lesions in MDMA users are more diffuse and less likely to follow a predictable anatomical distribution. However, early signs such as incisal edge wear, enamel pitting, and increased plaque retention may precede overt cavitation, particularly in the presence of bruxism and trismus commonly induced by MDMA use (26, 35).

The higher prevalence of posterior tooth wear in MDMA users likely reflects the intense clenching and grinding associated with bruxism during consumption. This pattern contrasts with the anterior maxillary wear reported in chronic MA users who snort the drug. Richard (32) hypothesized that this difference could be due to local vascular anatomy: The front upper teeth receive blood from the anterior and middle superior alveolar branches of the infraorbital artery, which branches off from the external carotid artery. These arteries also provide blood to the nasal lining. In contrast, the back upper teeth are supplied by the posterior descending branch of the sphenopalatine artery and the ascending palatine branches of the facial artery. Chronic constriction of the arteries supplying blood to the front upper teeth due to frequent snorting of MA could potentially result in reduced arterial blood flow to this region (33).

Experimental studies on the JOR in animals exposed to MDMA, though not directly translatable to humans, support clinical reports of jaw clenching and grinding lasting up to 48 h after consumption (8, 19, 25, 29, 32, 33, 35, 38). Some users adopt self-management strategies such as gum or pacifiers to reduce discomfort or protect teeth from excessive wear, though it may also contribute to certain patterns of dental erosion. Periodontitis in MA and MDMA users appear to be influenced both by the direct effects of the drug and by associated lifestyle factors, including poor oral hygiene, poly drug use and high consumption of tobacco and cannabis (14, 23, 29). Interestingly, smoking, although a recognized risk for periodontitis, appeared in one study (10) to have more pronounced effects on tooth decay than on periodontitis among MA users. This may be due to associated behaviors, such as higher sugary beverage intake or methods of drug use (ex: smoking) that exacerbate decay.

Dr. Nelson L. Rhodus (33) proposed that periodontal disease in stimulant users is largely linked to xerostomia. The combination of dry mouth, high intake of carbonated soft drinks to alleviate it, heavy plaque accumulation, and poor oral hygiene creates a high-risk environment for periodontal breakdown. Local ischemia from the vasoconstrictive properties of these drugs may also contribute, as illustrated by a case in which ecstasy storage in the labial vestibule led to localized periodontal damage (20). The variety of mucosal lesions observed in MDMA users suggests multiple pathogenic pathways. In some cases, direct trauma from bruxism or trismus appears to be the primary cause, as in the severe lower lip injury (29). In others, an acute inflammatory or immunogenic reaction may be implicated, the diffuse swelling reported in two separate cases (17, 37) could be explained by increased vascular permeability secondary to a drug-induced reaction on the vessel wall, resulting in edema and acute leukocytosis. This aligns with the possibility of an allergic response to MDMA or to one of its concomitant adulterants. However, purely mechanical causes such as frictional or masticatory trauma cannot be excluded, especially when swelling coexists with bruxism and ulcerations.

Interestingly, the oral health deterioration seen in MDMA, MA and polydrug users, marked by plaque accumulation, reduced salivary flow and frequent consumption of sugary beverages shares clinical similarities with Early Childhood Caries (ECC) (38, 39). These shared mechanisms highlight the importance of oral hygiene behaviors lifestyle regardless of age group can lead in promoting carious lesions. A comparative summary of oral manifestations across these groups is provided in Table 2.

**Table 2:** Table of comparison of oral manifestations outcomes in early childhood caries (ECC) MDMA, MA and polydrug use.

Aspect compared	Early childhood caries (ECC)	MDMA (Ecstasy)	Methamphetamine (MA)	Polydrug use
Presence of xerostomia	No, but decreased salivary flow in a context of breastfeeding during the night (38)	Yes, frequently reported (dose-dependent) (25, 36)	Yes, intense with a persistent dry mouth (9)	Yes, highly prevalent especially with the association psychotropic/ cannabis (23, 36)
Carious lesions	Rampant smooth-surface lesions in early life rapid progression linked to sugar exposure and poor hygiene (39)	Moderate to high linked to drink intake and xerostomia (36)	Very high severe “meth mouth” (9, 10), aseptic tooth pulp necrosis (40)	High to very high due to synergistic drug effect and poor oral care (14, 23, 25)
Tooth wear lesions	Rare	Evidence of tooth wear in the molar region from bruxism and little incisal wear (8, 31)	Pronounced wear including anterior teeth influenced by route administration (32)	Mixed patterns linked to bruxism, erosion, poor oral care (28)
Periodontitis	Occasional, mostly plaque related (39)	Twice higher prevalence compared to controls (36)	High prevalence: 89% in a study cohort (10)	Worsened by polydrug (23, 25)
Soft tissue lesions	Rare	Ulcerations, swelling, necrosis reported (17, 19)	Ulcerations and tissue damage secondary to trauma or neglect (9), hairy black tongue (40)	Common: ulcers, edema, necrosis due to multiple drug interactions (34)
Anatomical distribution	Primary maxillary incisors (39)	Diffuse, less predictable (8)	Vestibular surfaces of posterior teeth, progressing to coronal destruction (9, 40)	Variable, often generalized (23, 28, 34)
Oral hygiene	Often neglected by caregivers (39)	Reduced (25% brush <2×/day) (36)	Almost absent (9, 10)	Often poor (23, 34)

This review presents several limitations. Restricting publications in English and French, may exclude relevant data published in other languages. The available studies on the oral impact of MDMA are often heterogeneous in methodology, terms of design with small or non-representative samples which contributes to a high risk of bias and consequently limits the overall level of evidence. This disparity should be taken into account when interpreting the findings of this review. Moreover, few studies isolate the specific effects of MDMA without the co-use of other substances, making it difficult to attribute the observed oral consequences solely to MDMA. It is worth noting that several included studies in this review often grouped MDMA with MA and other MA type-stimulants due to their similar chemical structures and sympathomimetic effects that sometimes led to ambiguity.

This review provides a comprehensive and up-to-date synthesis of MDMA-related oral health effects, incorporating recent data up to 2025. It expands on previous works by integrating large-scale cross-sectional studies (36) and recent case reports documenting mucosal lesions and soft tissue destruction (19, 29). In addition, it offers a novel comparative framework including MDMA, MA, polydrug use and ECC (Table 2) providing a more precise and interdisciplinary understanding of the topic.

## 5 Conclusion

This narrative review highlights the wide range of oral health consequences associated with MDMA and MA use, from xerostomia and tooth decay to periodontitis, and soft tissue lesions. These effects arise from both drug-induced physiological changes and high-risk behaviors, often exacerbated by polydrug use and poor oral hygiene. Given the continuous emergence of new synthetic substances, ongoing surveillance and preventive strategies remain essential to protect oral health and clinicians have to be aware of the constant evolution of the drug components, the strength, the way of consumption and the consumers.

## Data Availability

The original contributions presented in the study are included in the article/Supplementary Material.
